# Hydrogen atom collisions with a semiconductor efficiently promote electrons to the conduction band

**DOI:** 10.1038/s41557-022-01085-x

**Published:** 2022-11-21

**Authors:** Kerstin Krüger, Yingqi Wang, Sophia Tödter, Felix Debbeler, Anna Matveenko, Nils Hertl, Xueyao Zhou, Bin Jiang, Hua Guo, Alec M. Wodtke, Oliver Bünermann

**Affiliations:** 1 Institute of Physical Chemistry, Georg-August University, Göttingen, Germany; 2 Department of Chemistry and Chemical Biology, University of New Mexico, NM, USA; 3 Department of Dynamics at Surfaces, Max-Planck-Institute for Multidisciplinary Sciences, Göttingen, Germany; 4 Hefei National Research Center for Physical Science at the Microscale, Department of Chemical Physics, University of Science and Technology of China, Hefei, China; 5 International Center of Advanced Studies of Energy Conversion, Georg-August University, Göttingen, Germany; 6 Present Address: Department of Chemistry, University of Warwick, Coventry, United Kingdom

**Keywords:** Energy transfer, Reaction kinetics and dynamics, Surface chemistry

## Abstract

The Born–Oppenheimer approximation is the keystone of modern computational chemistry and there is wide interest in understanding under what conditions it remains valid. Hydrogen atom scattering from insulator, semi-metal and metal surfaces has helped provide such information. The approximation is adequate for insulators and for metals it fails, but not severely. Here we present hydrogen atom scattering from a semiconductor surface: Ge(111)*c*(2 × 8). Experiments show bimodal energy-loss distributions revealing two channels. Molecular dynamics trajectories within the Born–Oppenheimer approximation reproduce one channel quantitatively. The second channel transfers much more energy and is absent in simulations. It grows with hydrogen atom incidence energy and exhibits an energy-loss onset equal to the Ge surface bandgap. This leads us to conclude that hydrogen atom collisions at the surface of a semiconductor are capable of promoting electrons from the valence to the conduction band with high efficiency. Our current understanding fails to explain these observations.

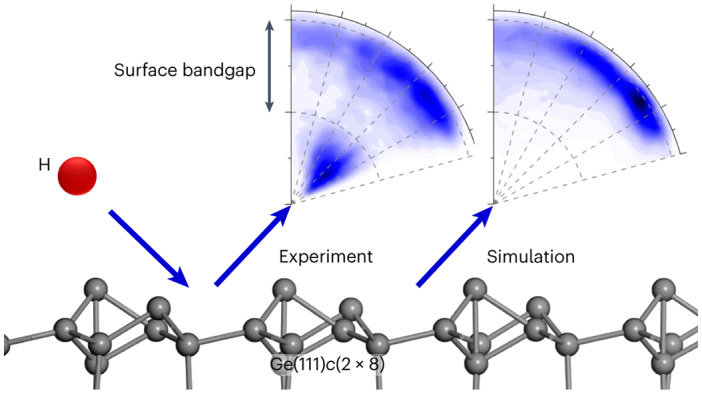

## Abstract (web summary)

Inelastic hydrogen atom scattering from surfaces provides a good benchmark for the validity of the Born–Oppenheimer approximation in surface chemistry. Now it has been shown that hydrogen atoms colliding with a semiconductor surface can efficiently excite electrons above the surface bandgap, representing a clear example of the failure of the approximation.

## Main

Atoms and molecules colliding at solid surfaces create time-varying electric fields that, due to their finite masses and associated low speeds, represent frequencies typically ≤10^13^ Hz, whereas much lighter electrons in solids oscillate at frequencies one to two orders of magnitude higher than this. This separation of timescales is used to justify the Born–Oppenheimer approximation (BOA)^1^, the bedrock of computational surface chemistry^2^, where electronic quantum states rapidly adjust to the motion of nuclei. Inelastic H atom surface scattering experiments have provided excellent benchmarks against which theoretical methods can and have been tested and proved^3^. Using this approach, the BOA has been shown to be justified for H atom scattering from Xe, where molecular dynamics (MD) simulations using a full-dimensional potential energy surface (PES) quantitatively reproduced energy losses measured in high-resolution scattering experiments^4^. The validity of the BOA in that case is not surprising since the lowest energy electronic excitations in Xe exceeded the energies of that work. Similar energy-loss measurements from experiments scattering H and D from the semi-metal graphene, where low-energy electron–hole pair (EHP) excitations are possible, also showed no signs of BOA failure^5–7^. Despite these successes, there are reasons to question the validity of the BOA (refs. ^8,9^). For example, energetic H atoms colliding at metal surfaces always excite EHPs (refs. ^10,11^). However, theoretical methods could successfully treat this with a weak-coupling ‘electronic-friction’ approximation^12,13^, suggesting BOA failure is not severe and can be accounted for in a perturbative fashion.

Experiments with semiconductors present an opportunity to make predictions from our current understanding about a fundamentally different class of solids. This is true if semiconductors behave in some hybrid fashion, reflecting some intermediate between insulators and metals. However, let us consider semiconductors from the point of view of another kind of time-varying electric field. We know visible light with electric fields oscillating at ~10^14−15^ Hz efficiently excites electrons from the valence band (VB) to the conduction band (CB), forming the basis for a large fraction of optical science and technology. This raises the question: if collisions of atoms and molecules with semiconductors could produce time-varying electric fields oscillating at similar frequencies, would they not also excite VB electrons to the CB and might this not provide important new avenues of research with the promise of new technology? If we were to adopt the physical picture derived from our study of metals, where electronic friction describes BOA failure, the answer to this question would certainly be ‘no’ or more precisely ‘only weakly’, as electronic-friction theories lead to hot EHP distributions that still favour low-energy excitation near the Fermi level^12^. Unfortunately, scattering experiments with semiconductors that test the validity of the BOA are rare. Transient currents were observed when Xe atoms with energies between 3 and 10 eV were scattered from surfaces of semiconductors^14–16^. However, this resulted from the creation of a local hot spot where initial phonon excitation subsequently transferred energy to EHPs. While these experiments provide us with clear evidence of BOA failure in a semiconductor, we can gain only little insight into the dynamics of the atom–surface collision. In fact, an electronically adiabatic model could describe the energy loss of scattered Xe atoms.

In the work presented in this article, we produce H atoms whose speeds are high enough to test the limits of the BOA directly by investigating the characteristics of their collisions with a semiconductor surface. The measured H atom energy-loss spectra and angular distributions reveal the excitations appearing in the solid on the sub-picosecond time scale. We find that, not only is VB–CB excitation possible, at sufficiently high energies it dominates the energy-transfer dynamics, showing that new physical mechanisms are at play. Specifically, we present translational energy-loss measurements on energetic H atoms scattered from a reconstructed Ge(111)*c*(2 × 8) surface along with first principles electronically adiabatic MD simulations, performed with a newly developed high-dimensional neural-network PES (NN-PES). When incidence energies are below the bandgap, only one scattering channel arises with small energy losses nearly identical to those seen in the MD simulations. These exhibit collision dynamics similar to those seen in H scattering from Xe. Surprisingly, at higher incidence energies, a second channel appears whose energy-loss onset is coincident with the semiconductor bandgap. This channel is absent in the MD simulations with and without electronic friction. The importance of this channel increases rapidly with H atom velocity—a signature of BOA failure—and accounts for ~90% probability at the highest H atom incidence energies of this work.

## Results

**Fig. 1:**
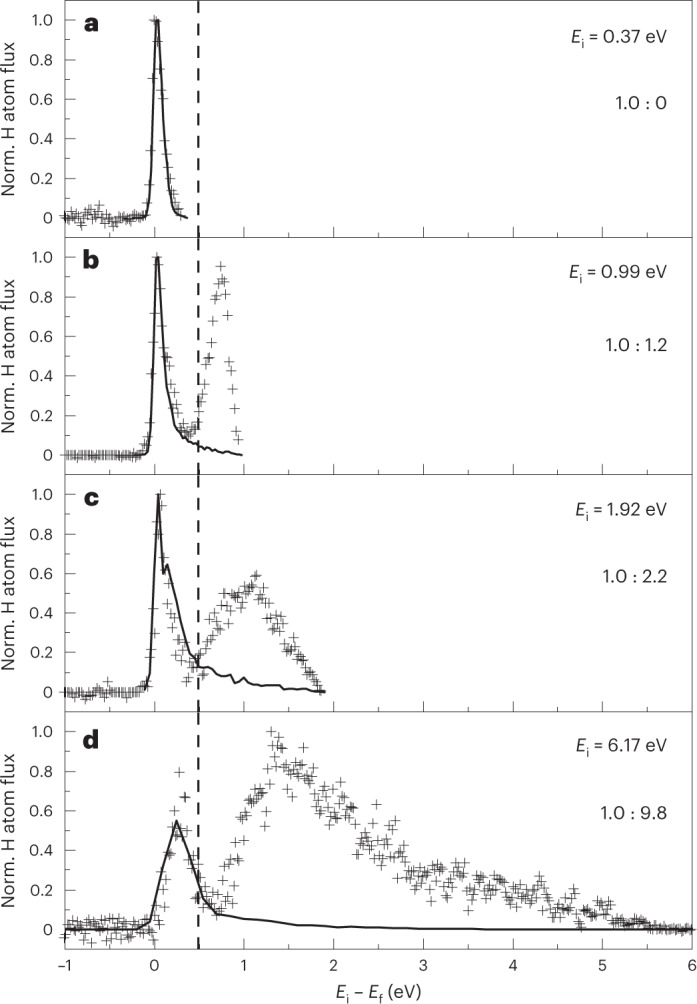
Translational energy-loss distributions for H atoms scattered from Ge(111)*c*(2 × 8). The incident H atoms travel along the [$\bar 110$] surface direction, while the polar incidence and scattering angles *ϑ*_i_ and *ϑ*_f_, respectively, were both 45° with respect to the surface normal. The surface temperature *T*_S_ was 300 K. **a**–**d**, Experimental data (+) and the results of adiabatic molecular dynamics simulations (solid lines) for four H atom translational incidence energies are shown: *E*_i_ = 0.37 eV (**a**), 0.99 eV (**b**), 1.92 eV (**c**) and 6.17 eV (**d**). The bandgap of the surface is 0.49 eV and is indicated by the vertical dashed line. The experimentally obtained ratio of the adiabatic to the VB–CB channel appears in each panel. All experimental curves are normalized to the peak intensity. The MD curves are scaled to fit the adiabatic channel. Source data

**Extended Data Fig. 1:**
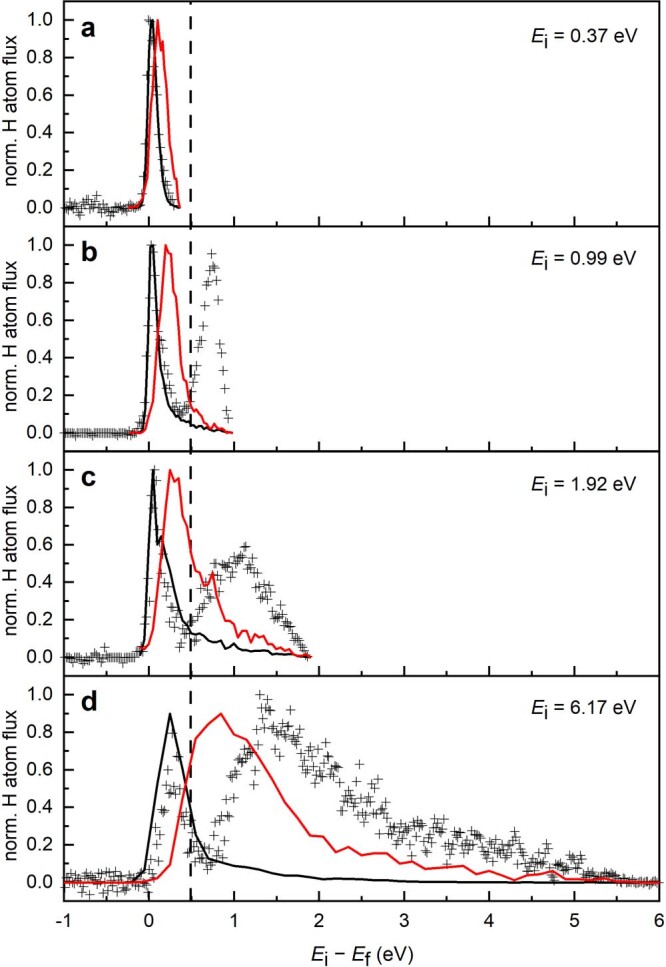
Comparison of molecular dynamics including electronic friction (EF) to adiabatic MD simulations and experiment. The incident H atoms travel along the [$\bar 110$] surface direction, while the polar incidence and scattering angles *ϑ*_i_ and *ϑ*_f_, respectively, were both 45° with respect to the surface normal. The surface temperature *T*_S_ was 300 K. **a-d**, Experimental data (+) as well as the results of adiabatic MD simulations (black solid lines) and MD simulations including EF (red solid lines) for four H atom translational incidence energies are shown: *E*_i_ = 0.37 eV (**a**), 0.99 eV (**b**), 1.92 eV (**c**) and 6.17 eV (**d**). The bandgap of the surface is 0.49 eV and indicated by the vertical dashed line. All experimental curves are normalized to the peak intensity. The MD curves are scaled to fit the experiment. Source data

Figure 1 shows experimental translational energy-loss distributions for H atoms scattered from Ge(111)*c*(2 × 8)^17^ at incidence energies *E*_i_ above and below the 0.49 eV surface bandgap^18^. We note that the given value for the surface bandgap was determined at a surface temperature of 30 K. However, a similar value is expected at room temperature since the reconstruction of the surface is unchanged. Also shown are the predictions of the electronically adiabatic MD trajectory calculations. Below the bandgap (Fig. 1a) only a single feature appears in the energy-loss distribution. The MD simulations reproduce the experimental result extremely well. MD simulations with electronic friction^19^ at the level of local density friction approximation (LDFA)^20^ fail to describe the energy-loss distributions (Extended Data Fig. 1). Analysis of adiabatic MD trajectories shows that H atoms interact with the Ge surface for only a few femtoseconds and that energy exchange is limited. Figure 1b–d shows energy-loss distributions for three values of *E*_i_ larger than the surface bandgap. In all three cases, the distributions are bimodal and the MD trajectories reproduce only the feature seen at low values of energy loss. Hereafter, we refer to this feature as the adiabatic channel. The second feature appearing at higher energy losses is absent in the adiabatic MD simulations, strongly suggesting that this channel involves conversion of H atom translational energy to electronic excitation of the Ge solid. This idea is further supported by the observation that the energy-loss onset of this feature is coincident (within experimental uncertainty) with the Ge surface bandgap of 0.49 eV at all values of *E*_i_. Furthermore, as expected for a channel involving BOA failure, this channel is strongly promoted by incidence translational energy, becoming about 90% of the observed scattering at the highest value of *E*_i_ = 6.17 eV. For these reasons, we assign the high energy-loss feature to an electronically non-adiabatic process where the collision of the H atom at the surface promotes an electron above the bandgap of the Ge surface. We refer to this mechanism hereafter as the VB–CB channel.

**Fig. 2:**
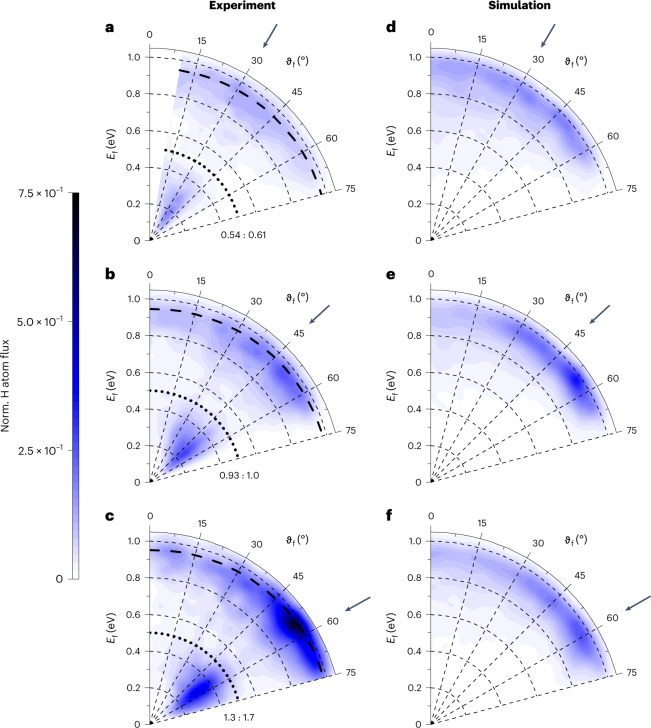
Incidence-angle dependence of H atoms scattered from Ge(111)*c*(2 × 8). Energy-resolved angular distributions derived from in-plane scattering flux are shown for three incidence angles, *ϑ*_i_ = 30, 45 and 60° and an incidence translational energy *E*_i_ = 0.99 eV. The surface temperature was *T*_S_ = 300 K. **a**–**f**, Experimental results (**a**–**c**) are compared to MD simulations (**d**–**f**). The adiabatic and the VB–CB channels both exhibit maximum scattering flux near the specular scattering angle (arrows). The MD simulations reproduce the behaviour of the adiabatic channel only. To construct the experimental plots, data were recorded in 5° increments from *ϑ*_f_ = 0 to 75°. All six polar plots are normalized to the incident H atom flux. The numbers show the ratios of the experimentally observed scattering channels with respect to the adiabatic channel for an incidence angle of *ϑ*_i_ = 45°: the left one corresponds to the VB–CB channel and the right one to the adiabatic channel. The MD simulations are scaled to experiment such that at an incidence angle of *ϑ*_i_ = 45°, the integrated adiabatic channels are equal in both. The black dashed lines represent the final energy predicted by a line-of-centres binary collision model: *E*_f_ **=** *E*_i_{1 − cos^2^[(*ϑ*_i_ + *ϑ*_f_)/2] × [1 − (*m*_H_ − *m*_Ge_)^2^/(*m*_H_ + *m*_Ge_)^2^]}. The black dotted lines indicate the surface bandgap of 0.49 eV. Source data

Figure 2 shows differential properties from both experiment and theory for H atoms incident at three angles *ϑ*_i_ and at *E*_i_ = 0.99 eV. Here, polar plots display the final translational energy *E*_f_ as a function of final scattering angle *ϑ*_f_. The black dotted lines show the expected minimal energy loss for excitation of an electron across the surface bandgap, which demarcates the adiabatic from the VB–CB channel. Experiment shows that the VB–CB channel exhibits a much narrower angular distribution (Table 1) than the adiabatic channel at all three incidence angles. The MD simulations yield similar differential scattering maps as seen in experiment for the adiabatic channel only. The energy loss agrees with experiment and even the experimentally observed dependency of the angular distribution on *ϑ*_i_ is reproduced. The VB–CB channel is absent in the MD simulations.

**Table 1:** Angular full width at half maximum for the experimental angular distributions of this work

*E*_i_	VB−CB			Adiabatic		
	*ϑ*_i_ = 30°	*ϑ*_i_ = 45°	*ϑ*_i_ = 60°	*ϑ*_i_ = 30°	*ϑ*_i_ = 45°	*ϑ*_i_ = 60°
0.99 eV	24°	31°	24°	>56°	44°	34°
1.92 eV	−	>70°	−	−	>73°	−

**Fig. 3:**
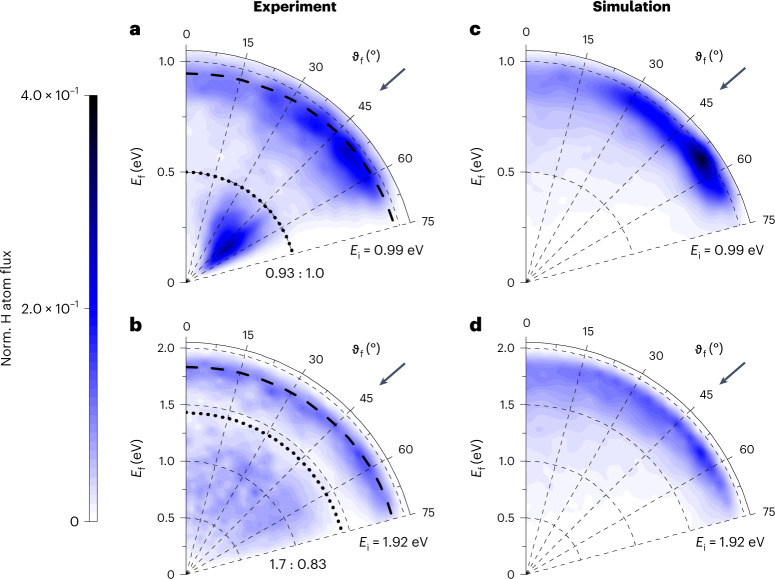
Incidence-energy dependence of H atoms scattered from Ge(111)*c*(2 × 8). **a**–**d**, Energy-resolved angular distributions derived from in-plane scattering flux are shown for two incidence translational energies *E*_i_ = 0.99 eV (**a** and **c**) and 1.92 eV (**b** and **d**). The surface temperature was *T*_S_ = 300 K and the incidence angle is *ϑ*_i_ = 45°. Experimental results (**a** and **b**) are compared to MD simulations (**c** and **d**). The MD simulations reproduce the behaviour of the adiabatic channel only. To construct the experimental plots, data were recorded in 5° increments from *ϑ*_f_ = 0 to 75°. All four polar plots are normalized to the incident H atom flux. The numbers show the ratios of the experimentally observed scattering channels with respect to the adiabatic channel for an incidence energy of *E*_i_ = 0.99 eV: the left one corresponds to the VB–CB channel and the right one to the adiabatic channel. The MD simulations are scaled to experiment such that at an incidence energy of *E*_i_ = 0.99 eV, the integrated adiabatic channels are equal in both. The black dashed lines represent the final energy predicted by a line-of-centres binary collision model: *E*_f_ = *E*_i_{1 − cos^2^[(*ϑ*_i_ + *ϑ*_f_)/2] × [1 − (*m*_H_ − *m*_Ge_)^2^/(*m*_H_ + *m*_Ge_)^2^]}. The black dotted lines indicate the surface bandgap of 0.49 eV. Source data

Figure 3 shows polar plot representations similar to Fig. 2 emphasizing the incidence energy dependence of the scattering. As before, the experimental results show bimodal scattering distributions with two well-resolved channels separated in energy space by the bandgap energy, marked as black dotted lines. The angular distributions of both channels broaden between *E*_i_ = 0.99 and 1.92 eV, but the VB–CB channel broadens significantly more as it is narrower at *E*_i_ = 0.99 eV (Table 1). The adiabatic MD simulations (Fig. 3c,d) reproduce this effect for the adiabatic channel.

The average energy losses derived from the experiments are summarized in Table 2. Note that for the adiabatic channel, the average energy transferred to the surface *<E*_i_ − *E*_f_ > is a small and nearly constant fraction (10 ± 5%) of *E*_i_. The VB–CB channel behaves differently, as the fraction of incidence energy transferred to the solid goes up dramatically as *E*_i_ is reduced. This is an influence of the surface bandgap, where the absolute of energy lost must exceed 0.49 eV, regardless of *E*_i_. Hence, at lower values of *E*_i_ the fractional energy loss must sharply increase. Note also that the average energy lost decreases only slightly with increasing *ϑ*_i_ for both channels.

**Table 2:** Average energy-loss in experimentally obtained specular (*ϑ*_i_ = *ϑ*_f_) H atom scattering

		VB−CB		Adiabatic	
*E*_i_	*ϑ*_i_	〈*E*_i_ − *E*_f_〉	$\frac{{\left\langle {E_{{{\mathrm{i}}}} - E_{{{\mathrm{f}}}}} \right\rangle }}{{E_{{{\mathrm{i}}}}}} \times 100$	〈*E*_i_ − *E*_f_〉	$\frac{{\left\langle {E_{{{\mathrm{i}}}} - E_{{{\mathrm{f}}}}} \right\rangle }}{{E_{{{\mathrm{i}}}}}} \times 100$
0.37 eV	45°	−	−	0.05 eV	14% (13%)
0.99 eV	30°	0.75 eV	75%	0.15 eV	15% (17%)
	45°	0.71 eV	72%	0.13 eV	13% (12%)
	60°	0.69 eV	70%	0.10 eV	10% (8.1%)
1.92 eV	45°	1.12 eV	58%	0.20 eV	10% (14%)
6.17 eV	45°	2.28 eV	37%	0.32 eV	5.2% (7.7%)

Values in parentheses were computed from adiabatic MD trajectories

## Discussion

We start by highlighting some of the key observations just presented and their implications. First, Fig. 2 shows clearly that the most probable value of *ϑ*_f_ depends on the chosen value of *ϑ*_i_, proving the scattered atoms did not thermalize with the solid. Thermalization occurs on the picosecond timescale. Thus, we conclude that the scattered atoms in both channels experience a sub-picosecond interaction time with the surface. Second, there is evidence of sticking, even though integrated scattering probabilities such as sticking probabilities cannot be easily obtained from in-plane differential scattering measurements, since the fraction of incident atoms that scatter out of the detection plane may also depend on incidence conditions and branching channel. We can nevertheless integrate the observed scattering at flux over *E*_f_ and *ϑ*_f_. These integrals scaled to the experimentally observed adiabatic channel at *E*_i_ = 0.99 eV and *ϑ*_i_ = 45° appear as numbers next to each differential scattering diagram in Figs. 2 and 3. They are given as ratios that report the relative contributions of the two scattering channels. There is an overall loss of signal between *E*_i_ = 1.92 and 0.99 eV. If we were to assume the out-of-plane scattering fraction was independent of *E*_i_, we would conclude that the sticking probability decreases with increasing incidence energy. A similar trend is seen in the MD simulations. Note also that the branching ratios shown in Fig. 3a,b are consistent with those of Fig. 1b,c, which represent the branching between the two scattering channels detected at *ϑ*_f_ = 45° only. This agreement suggests that the branching seen in Fig. 1c (*E*_i_ = 1.92 eV) is representative of other scattering angles.

The major outcome of this work is the observation that an H atom scattering from a semiconductor may experience one or the other of two types of interaction, either a mechanical interaction well described within the BOA or a strong non-adiabatic interaction capable of promoting an electron to energies above the bandgap. We emphasize that while there are similarities with past work, the behaviour seen here is qualitatively different from previous observations involving insulators, metals or semi-metals. For example, the adiabatic channel seen in Figs. 1–3 exhibits marked similarities to H atom scattering from insulating Xe. However, that system exhibited no BOA failure whatsoever. Conversely, H scattering trajectories describing collisions with metals simultaneously excite both phonons and EHPs (refs.^10–13^), the two excitations being inextricably linked to one another. The question remains, what gives rise to the branching between the two channels in the H/Ge system?

The fact that H scattering from Ge exhibits branching behaviour between two distinct dynamical channels is consistent with a two-state picture. We envision that the H atom proceeds initially along the ground electronic state until it encounters a seam of crossing associated with a short-lived electronically excited state. (Note that the word state is used here loosely as many electronic states are involved in the VB and CB of the system.) We assume that this state rapidly decays into unoccupied electronic states within the CB. At low incidence energies, reaching the seam of crossing requires specific approach, but at higher energies other regions of the seam become accessible with reduced steric restrictions.

Evidence supporting this picture can be found in observations of this work, especially Fig. 2. Note that the VB–CB channel exhibits a narrow angular distribution, peaking near the specular scattering angle (arrows in Fig. 2). This shows that there is no preference for loss of incidence energy parallel or perpendicular to the surface when inducing electronic excitation. A narrow angular distribution is typical of scattering influenced by directional forces associated with atomic orbitals with preferred orientations, which is consistent with the suggested mechanism of a curve crossing, where H atom collisions must occur at specific surface sites (Ge atoms) and with specific approaching geometries. Figure 3 shows that at a higher energy these steric restrictions appear to be less severe and consequently the VB–CB scattering angular distribution broadens.

Contrasting this behaviour, the adiabatic channel exhibits a markedly broader angular distribution even at low incidence energies. This indicates a large corrugation of the PES experienced by the atoms passing through the adiabatic channel. Despite the many final scattering angles, the energy loss follows a hard-sphere line-of-centres binary collision model (black dashed lines). This indicates that the H atom scattered through the adiabatic channel is experiencing binary collisions with many impact parameters. It is not surprising, due to the complex surface structure of the Ge(111)*c*(2 × 8) surface, if the H atoms scattering through the adiabatic channel sample a large fraction of the surface unit cell.

Bimodal energy-loss distributions may be produced without electronic excitation. For example, H scattering from a graphene layer involves trajectories that either fail or succeed in surmounting a chemisorption barrier^5–7^. H atoms reflected from the barrier experience weak van der Waals interactions with little energy transferred, while H atoms surmounting the barrier couple strongly to in-plane phonons of the graphene layer^5^. In contrast to this behaviour, the electronically adiabatic MD simulations carried out in this work show no sign of bimodal distributions. This is consistent with the absence of a chemisorption barrier in the H/Ge system. The combined strength of the experimental and theoretical results supports our assignment of an electronically adiabatic and a non-adiabatic channel.

While it is common knowledge that absorption of photons in the bulk of a semiconductor excites electrons from the VB to the CB, this work shows that a colliding atom may efficiently promote electrons in a similar way in a purely surface-specific process. The probability to convert translational energy of the H atom to electronic excitation of the solid dramatically increases with incidence energy, as does the average excitation energy. The large excitation probability as well as the large energy loss is inconsistent with electronic-friction theories. Hence, this work stands as a challenge for new theories of electronically non-adiabatic surface chemistry. We hasten to add that the designation of this behaviour as VB–CB represents a simplified viewpoint. The precise nature of the excited electronic states involved is still unknown. Transient surface-localized excitations (even plasmons) might be important. Nevertheless, the observation that electronic excitation dominates the dynamics in collisions of a simple atom with a semiconductor opens new horizons for research into non-adiabatic effects in surface chemistry and chemical sensors.

## Methods

The experimental setup is described in detail in refs. ^3,21^. Briefly, ultraviolet (*λ*_photolysis_ = 248.35 nm) or vacuum ultraviolet (*λ*_photolysis_ = 121.4 nm) photodissociation of a supersonic molecular beam of hydrogen iodide produces a H atom beam with translational energies of *E*_i_ = 0.37, 0.99, 1.92 or 6.17 eV that then passes through two differential pumping chambers to enter an ultra-high vacuum scattering chamber before colliding with a germanium crystal. The Ge sample is held on a five-axis manipulator, allowing the variation of the polar incidence angle *ϑ*_i_ with respect to the surface normal. The scattered H atoms are excited to a long-lived Rydberg state just below the ionization limit^22^ and fly 250 mm before they are field ionized and detected by a multichannel plate assembly. A multichannel scaler records the arrival time to obtain the time-of-flight distributions, which we convert to energy spectra by applying the appropriate Jacobians. The detector is rotatable in the plane defined by the incident H atom beam and the surface normal allowing time-of-flight distributions to be obtained at various final scattering angles *ϑ*_f_. The used Ge crystal is undoped with a purity of 99.999%. The Ge(111) surface was cleaned with cycles of Ar^+^ ion sputtering and annealing to ~670 °C. Auger electron spectroscopy (AES) and low-energy electron diffraction (LEED) validated the cleanliness and *c*(2 × 8) structure of the surface.

To perform theoretical simulations, a neural-network PES (NN-PES) was constructed for the H@Ge(111)*c*(2 × 8) system and MD simulations were performed. Data for the NN fitting were obtained with spin-polarized DFT calculations, carried out with the Vienna Ab initio Simulation Package (VASP)^23,24^ with the frozen-core all-electron projector-augmented wave (PAW) method^25,26^. The electronic wave function was expanded using plane waves with an energy cutoff of 250 eV. The electron exchange-correlation energies were described by the Perdew-Burke-Ernzerhof (PBE) functional within the generalized gradient approximation (GGA)^27^. The reconstructed Ge(111)*c*(2 × 8) surface was modelled by repeated slabs separated by a vacuum space of 16 Å in the *z* direction. Each slab contained eight atomic layers, with four additional Ge adatoms added on top of the first layer. The Ge atoms in the bottom layer not seen by the scattering H atoms in the MD simulations were capped by Ge–H bonds. The Ge adatoms and top six layers were allowed to move while the remaining atoms were fixed throughout the calculations. Therefore, there were a total of 101 movable atoms in the unit cell. The Brillouin zone was sampled with a 3 × 1 × 1 *k*-point grid. Ab initio molecular dynamics (AIMD) trajectories were used to provide training data for the NN fitting. The AIMD trajectories employed initial positions of the H atom randomly sampled 6 Å above the surface. About 100 AIMD trajectories were run for H atoms with incidence energies of 0.99 and 1.92 eV, an incidence angle of 45° and a surface temperature of 300 K, providing ~150,000 points. Additional single-point DFT calculations were performed to augment the AIMD points. The data set were culled using a Euclidean distance of 0.3 Å to remove points that were too close to one another. About 26,000 points (including both energy and gradient) were finally selected to fit a 303-dimensional PES using an embedded-atom neural network (EANN) approach^28^. The EANN PES obtained in this way was thoroughly tested, giving a root-mean-square error (RMSE) of about 80 meV per cell (or 0.8 meV per atom). MD trajectories were calculated with a modified Venus program^29^. The timesteps were chosen separately for each incidence energy: 0.10, 0.05, 0.03 and 0.01 fs for 0.37, 0.99, 1.92 and 6.17 eV, respectively.

To study possible non-adiabatic effects, an electron-friction model was applied^19,30^. The electronic-friction coefficient was calculated based on the local-density friction approximation (LDFA)^20,31^. The electron density of the Ge(111)*c*(2 × 8) surface was obtained from about 100 configurations at 300 K. To obtain an analytical expression for the electron density the data were again fitted with the EANN method.

## Online content

Any methods, additional references, Nature Portfolio reporting summaries, source data, extended data, supplementary information, acknowledgements, peer review information; details of author contributions and competing interests; and statements of data and code availability are available at 10.1038/s41557-022-01085-x.

## Data Availability

Source data are provided with this paper.

## Source data

Source Data Fig. 1 Source data.
Source Data Fig. 2 Source data.
Source Data Fig. 3 Source data.
Source Data Extended Data Fig. 1 Source data.

## Code availability

The EANN code of B.J. is available at https://github.com/zhangylch/REANN. The VENUS code is available at https://www.depts.ttu.edu/chemistry/Venus/index.php.
